# Statin use and the risk of hepatocellular carcinoma among patients with chronic hepatitis B: an emulated target trial using longitudinal nationwide population cohort data

**DOI:** 10.1186/s12876-023-02996-w

**Published:** 2023-10-25

**Authors:** Dong Hyun Sinn, Danbee Kang, Yewan Park, Hyunsoo Kim, Yun Soo Hong, Juhee Cho, Geum-Youn Gwak

**Affiliations:** 1 Department of Medicine, Samsung Medical Center, Sungkyunkwan University School of Medicine, 81 Irwon-ro, Gangnam-Gu, Seoul, 06351 South Korea; 2 Department of Clinical Research Design and Evaluation, SAIHST, Sungkyunkwan University, 81 Irwon-ro, Gangnam-Gu, Seoul, 06351 South Korea; 3 Center for Clinical Epidemiology, Samsung Medical Center, Sungkyunkwan University, Seoul, South Korea; 4 Department of Internal Medicine, Kyung Hee University Hospital, Seoul, Korea; 5 Departments of Epidemiology and Medicine, Welch Center for Prevention, Epidemiology and Clinical Research, Johns Hopkins Medical Institutions, Baltimore, MD USA

**Keywords:** Chronic Hepatitis B, Hepatocellular carcinoma, Statin

## Abstract

**Background:**

No randomized controlled trials have been completed to see whether statin can decrease hepatocellular carcinoma (HCC) risk in chronic hepatitis B (CHB) patients. We used large-scale, population-based, observational data to emulate a target trial with two groups, statin user and statin non-user.

**Methods:**

Among 1,379,708 nonunique individuals from the Korean National Health Insurance Service data, 2,915 CHB patients with serum cholesterol level of 200 mg/dL or higher who started statin therapy and 8,525 propensity-score matched CHB patients with serum cholesterol level of 200 mg/dL or higher who did not start statin therapy were analyzed for the development of HCC. In addition, liver cancer or liver-related mortality and all-cause mortality were assessed.

**Results:**

During follow-up, 207 participants developed HCC. Incidence rate of HCC was 0.2 per 1,000 person-years in the statin user group and 0.3 per 1,000 person-years in the statin non-user group. Fully adjusted hazard ratio (HR) for incident HCC comparing statin user group to statin nonuser group was 0.56 (95% confidence interval [CI]: 0.39 to 0.80). The association between statin use and decreased HCC risk was consistent in all subgroups analyzed. Fully adjusted HR comparing statin user to statin nonuser was 0.59 (95% CI: 0.35 to 0.99) for liver cancer or liver-related mortality and 0.93 (95% CI: 0.78 to 1.11) for all-cause mortality.

**Conclusions:**

Statin might have a benefit for preventing HCC in CHB patients with elevated cholesterol levels. Statin should be actively considered for CHB patients with dyslipidemia.

## Introduction

Chronic hepatitis B virus (CHB) infection is a major global health burden due to high mortality and morbidity of hepatitis B virus (HBV)-related liver disease and liver cancer [1–3]. Nowadays, antiviral therapies for HBV which can decrease the risk of cirrhosis complications and hepatocellular carcinoma (HCC) are available [4, 5]. However, current antiviral therapy for HBV cannot completely eliminate the risk of disease progression [4, 5]. An epidemiologic study has shown increased burden of liver cancer in HBV-endemic areas despite extensive use of antiviral therapy [4]. There is a clinically unmet need to develop additional chemo-preventive strategies to reduce the risk of HCC in patients with CHB.

Statins are 3-hydroxy-3-methylglutaryl CoA (HMG-CoA) reductase inhibitors with a long history of use in treatments for dyslipidemia and cardiovascular disease [6]. Beside of their cardioprotective effect, statins have pleiotropic properties with possible chemo-preventive effects [7, 8]. Meta-analysis of observational studies has found a reduced risk of HCC with statin use compared to no statin use [9, 10]. Yet, randomized controlled trials (RCTs) assessing chemo-preventive effect of statin on incident HCC in CHB patients have not been reported yet. There are numerous examples of studies showing that some therapies seem to be effective when they are investigated by observational methods, but later found to be contradicted by evidence from RCTs, and vice versa [11]. In lung cancer, meta-analysis of observational studies has shown an improvement of overall survival by statin use compared to no statin use, but meta-analysis of RCTs could not find any association [12]. RCTs are required to confirm risks and benefits of statin therapy for its chemo-preventive indication for HCC in CHB patients. However, RCTs require enormous resources including medical care, patients’ participation, human resources, and time to observe events. An emulated target trial using observational data is a method to help avoid methodologic pitfalls in observational studies [13]. It can yield the same effect estimate as that of a RCT if the emulation is successful [14]. It can also provide timely information.

HBV infection is the most common cause of chronic liver disease and HCC in South Korea [2, 15]. South Korea has a government-mandated universal health insurance coverage for the entire population [16]. The Korean National Health Insurance Service (K-NHIS) collects all claims for inpatient and outpatient visits, procedures, and prescriptions in the K-NHIS database and provide a standardized national health screening to all insured people [17–20]. Using this large-scale, population based observational data, we conducted an emulation trial to determine chemo-preventive effect of statin on HCC development in CHB patients.

## Methods

### Data sources

We performed a trial emulation study using the K-NHIS database. The K-NHIS database represents the entire population of South Korea. K-NHIS claims for inpatient and outpatient visits, procedures, and prescriptions were coded using the International Classification of Diseases, 10th Revision (ICD-10) [17]. As the K-NHIS routinely audits claims, such data are considered reliable and used in numerous peer-reviewed publications [18, 19]. In Korea, a standardized national health screening program is provided to all insured persons by K-NHIS every two years [20]. The national health screening includes a self-administered questionnaire on medical history and health behaviors, anthropometric measurements, and laboratory tests [20]. The participation rate among the target population is approximately 76% [20]. The review committee of the K-NHIS approved this study for the use of the K-NHIS and national health screening data (protocol number: NHIS-2021-1-575). The Institutional Review Board of the Samsung Medical Center approved the study and waived the requirement for informed consent because K-NHIS data were de-identified data.

### Study population

We included adults who met all of the following inclusion criteria at the index visit between January 1, 2009 and December 31, 2017: (1) age between 40 and 84 years; (2) chronic HBV infection defined by ICD-10 code for chronic HBV infection (B18.0, B18.1, B18.18 or Z22.5) during the study period; (3) total cholesterol level of 200 mg/dL or higher; (4) no prescription code for statins for the prior six months. Then, we excluded participants who had at least 1 following condition: (1) history of cancer (ICD-10 code: C), (2) co-infection with human immunodeficiency virus (ICD-10 codes: B20, B21, B22, B24) or hepatitis C virus (ICD-10 code: B18.2), (3) history of liver cirrhosis (ICD-10 codes: K70.2, K70.3, K71.7, K76.1, K74), jaundice (ICD-10 code: R17), ascites (ICD-10 code: R18), or hepatic failure (ICD-10 code: K720), and (4) history of myocardial infarction (ICD-10 codes: I21-23, I252), stroke (ICD-10 codes: I60-63), heart failure (ICD-10 codes: I110, I130, I1132, I255, I420, I425-429, I43, I50, I971), coronary heart disease (ICD-10 codes: I43, I50, I099, I110, I130, I132, I255, I420, I425-429, P290), or revascularization (PCI, Korean National Health Insurance codes M6551-6554, M6561-6567, M6571-6572; coronary artery bypass graft surgery [CABG], O1640-1642, O1647-1649, OA640-642, OA647-649). The index visit was the time of national health screening exam, which included information on total cholesterol level.

### Measurements

The exposure was statin use. We identified all lipophilic statins (atorvastatin, simvastatin, fluvastatin, lovastatin, and cerivastatin) and hydrophilic statins (rosuvastatin and pravastatin) prescription code in the K-NHIS database after the index visit. We performed an intention to treat analysis. Subjects were assigned into a statin user group if the prescription was at least one day.

The primary endpoint was development of HCC during follow-up. HCC was defined as the presence of cancer-specific insurance claim code (V193 code) with a C22.0 code which was an ICD-10 code for HCC. Secondary endpoints were liver cancer or liver-related mortality, defined by presence of C22, B15-19, K70-75 codes in the death certificate, and all-cause mortality. In addition, we defined extrahepatic cancer-related mortality when the cause of mortality was cancer other than liver cancer (C code except for C22). Cardiovascular disease-related mortality was defined when the cause of mortality was cardiovascular diseases by following ICD-10 codes (I69, I63, I62, I61, I60, I50, I48, I46, I35, I34, I26, I25, I21, I20, I11, I10).Vital status and cause of death were obtained from death certification collected by Statistics Korea, part of the Ministry of Strategy and Finance of South Korea [18].

For covariates, the following information were collected: age, sex, body mass index (BMI), drinking status, smoking status, physical activity per week, laboratory variables (serum total cholesterol, high-density lipoprotein [HDL] cholesterol, low-density lipoprotein [LDL] cholesterol, aspartate aminotransferase [AST], alanine aminotransferase [ALT], gamma-glutamyl transferase [GGT]), systolic blood pressure, diastolic blood pressure, hypertension (ICD-10 codes: I10-13, I15), diabetes (ICD-10 codes: E11-14), and concomitant medications. For concomitant medication, information on other lipid lowering drugs (e.g., fibrate), calcium channel blocker, angiotensin-converting enzyme inhibitor, angiotensin II receptor blockers, beta-blocker, warfarin, aspirin, and anti-HBV medication were collected. Concomitant medications were defined if the patient had prescription identified based on Korean Drug and Anatomical Therapeutic Chemical Codes for the medication at 90 days prior to the index date.

### Assigned groups and follow-up

We emulated a pragmatic sequence of trials or pseudo-trials by aligning the eligibility window, treatment assignment, and start of follow-up between the statin user and statin nonuser groups [21]. To emulate a trial to determine the effect of statin on clinical outcome in patients with CHB, we identified CHB patients who met the eligibility criteria on the day of health screening visits and followed them until incident HCC, death, or December 2019, whichever occurred first. We classified participants into the statin user group if they used statin during a week which was the enrollment period. Otherwise, participants were assigned into the statin nonuser group. Next, using an approach as described previously [22], we emulated a second trial with baseline a week after the first trial, and so on for every week. In each trial, participants who met the exclusion criteria, those who were assigned to the statin user group in the previous trial, and those who developed an event (study endpoints) were excluded. All others were then re-assessed for statin use in the next seven days and classified into the two groups according to statin use. We repeated the entire process for health screening between date 0 and 180 days including all available health screening visits. Each participant could contribute as an eligible individual to as many trials as he or she was eligible. Since participant could had incidence of event and then took statin even within a week, we emulated a series of trials with a one-week enrollment period. Thus, participants who had outcome within a week were excluded. We followed them until the end date of a trial. Emulation of sequential trials is a valid and efficient procedure if participants meet eligibility criteria at several time points [23].

To reduce computational time, we used a 10% random subsample in the statin nonuser group. Propensity score (PS) matching was performed to minimize potential impact of confounders on outcomes. In the analysis, covariate variables were updated at the start of each trial. Multivariable logistic regression estimated the PS for the statin user group using the following variables at each trial entry date: all covariates in Table 1 and year-month at the entry of the trial. Matching was performed using a greedy algorithm (caliper was 0.15). Statin nonuser group was matched 3:1 with statin user group within each trial. In the analysis, we pooled data from all trials into a single model and included day at the trial’s baseline. A standardized mean difference (SMD) between statin user and nonuser groups was estimated to compare the distribution of variables used for matching.

**Table 1:** Baseline characteristics of study subjects

	Statin nonuser (*N* = 8,525)	Statin user (*N* = 2,915)	*P* value	SMD
**Age, years**	55.85 (9.31)	56.33 (8.92)	0.013	0.054
**Sex, male**	4,058 (47.6)	1,346 (46.2)	0.19	-0.029
**BMI**			0.506	
Underweight (< 18.5 kg/m^2^)	107 (1.3)	30 (1.0)		-0.021
Normal (18.5–23 kg/m^2^)	2,185 (25.6)	723 (24.8)		-0.019
Overweight (23–25 kg/m^2^)	2,134 (25.0)	711 (24.4)		-0.015
Obesity (> 25 kg/m^2^)	4,095 (48.0)	1,449 (49.7)		0.033
Unknown	4 (0.0)	2 (0.1)		0.009
**Drinking**			0.169	
None	5,108 (59.9)	1,799 (61.7)		0.037
Moderate	3,052 (35.8)	1,002 (34.4)		-0.030
Heavy	313 (3.7)	105 (3.6)		-0.004
Unknown	52 (0.6)	9 (0.3)		-0.045
**Smoking**			0.564	
None	5,260 (61.7)	1,828 (62.7)		0.021
Past	1,342 (15.7)	452 (15.5)		-0.006
Current	1,906 (22.4)	632 (21.7)		-0.016
Unknown	17 (0.2)	3 (0.1)		-0.025
**Physical activity (per week)**			0.016	
None	6,753 (79.2)	2,366 (81.2)		0.049
Regular	1,730 (20.3)	543 (18.6)		-0.042
Unknown	42 (0.5)	6 (0.2)		-0.049
**Total cholesterol, mg/dl**	233.20 (77.15)	243.09 (34.30)	< 0.001	0.166
**HDL cholesterol, mg/dl**	57.23 (25.60)	56.71 (27.30)	0.347	-0.020
**LDL cholesterol, mg/dl**	147.30 (80.62)	154.99 (44.42)	< 0.001	0.118
**AST, U/L**	32.69 (37.57)	31.85 (26.25)	0.264	-0.026
**ALT, U/L**	35.29 (56.85)	34.09 (33.27)	0.281	-0.026
**GGT, U/L**	54.23 (106.98)	50.09 (75.57)	0.054	-0.045
**Systolic blood pressure, mmHg**	126.11 (15.30)	126.53 (16.41)	0.205	0.027
**Diastolic blood pressure, mmHg**	78.73 (10.20)	78.79 (10.50)	0.792	0.006
**Hypertension (%)**	3,822 (44.8)	1,442 (49.5)	< 0.001	0.093
**Diabetes (%)**	3,331 (39.1)	1,232 (42.3)	0.003	0.065
**Concomitant medication**				
Other lipid lowering drug (%)	10 (0.1)	12 (0.4)	0.004	0.057
Calcium channel blocker (%)	215 (2.5)	130 (4.5)	< 0.001	0.106
ACEI (%)	29 (0.3)	21 (0.7)	0.012	0.052
Aspirin (%)	116 (1.4)	96 (3.3)	< 0.001	0.128
ARB (%)	159 (1.9)	118 (4.0)	< 0.001	0.129
Beta-blocker (%)	244 (2.9)	115 (3.9)	0.005	0.06
Warfarin (%)	4 (0.0)	1 (0.0)	> 0.999	-0.006
Antiviral medications (%)	85 (1.0)	29 (1.0)	> 0.999	0

Values are presented as n (%) or mean (SD).

*ACEI* angiotensin converting enzyme inhibitor, *ALT* alanine aminotransferase, *ARB* angiotensin II receptor blocker, *AST* aspartate aminotransferase, *BMI* body mass index, *HDL* high-density lipoprotein, *LDL* low-density lipoprotein, *GGT* gamma-glutamyl transferase, *SMD* standard mean difference

### Statistical analysis

The primary analysis was intention-to-treat analysis. Cumulative incidence of each outcome was estimated with the Kaplan-Meier method. Log rank tests were applied to evaluate differences between groups. We calculated hazard ratios (HR) with 95% confidence intervals (CI) for incidence of clinical outcomes using a Cox regression model. Covariates with SMD ˃ 0.1, which could provide evidence of an imbalance between matched groups, were adjusted for survival analysis. We examined proportional hazards assumption using plots of the log(-log) survival function and Schoenfeld residuals. All *P-*values were two-sided and a *P*-value of less than 0.05 was considered significant. All analyses were performed using SAS® Visual Analytics (SAS Institute Inc., USA) and R 4.1.2 (R Foundation for Statistical Computing, Vienna, Austria).

## Results

**Fig. 1:**
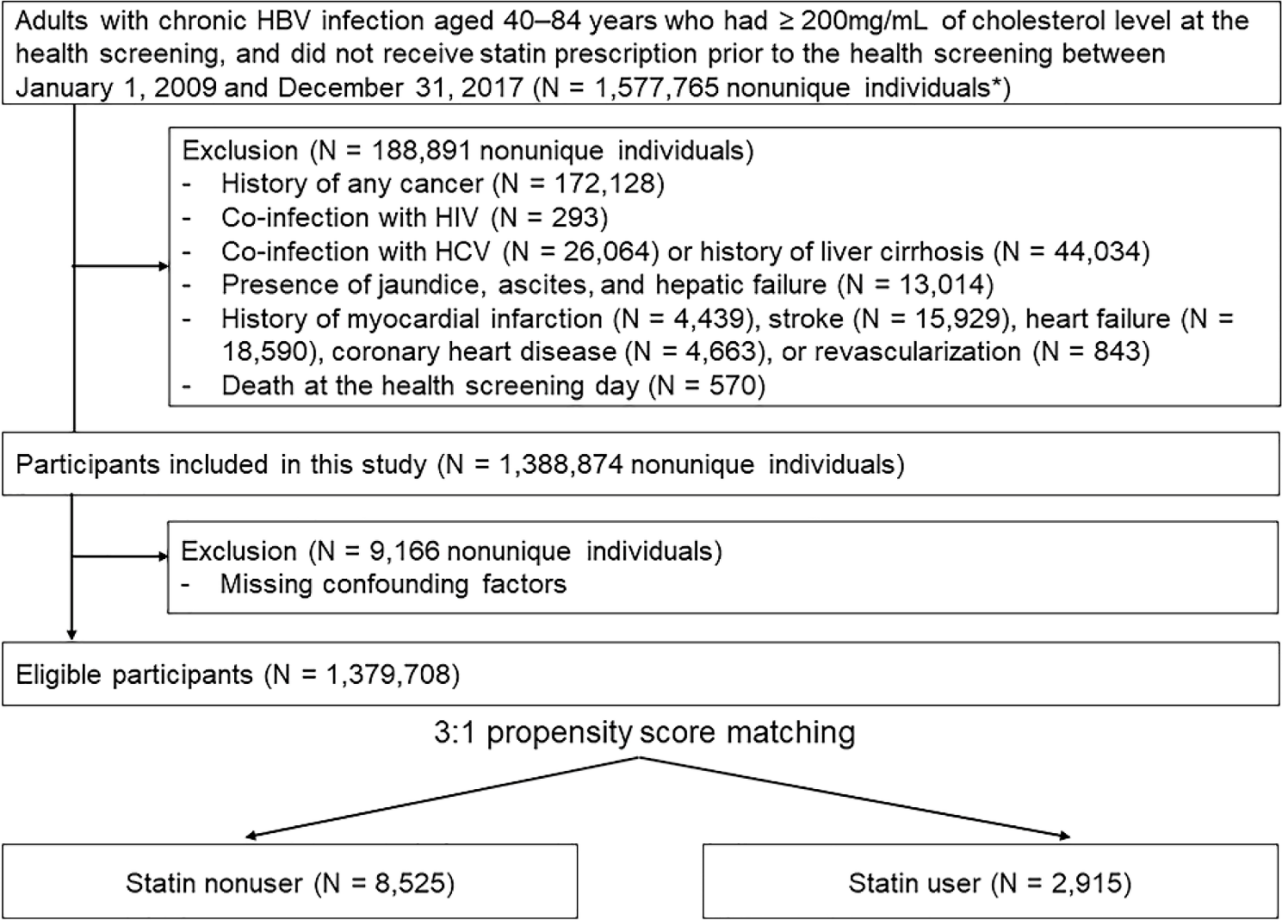
Flow chart of study subjects. Study participants could have more than one exclusion criteria. *10% random sampling was applied to the statin nonuser group

During the study period, 1,388,874 person-trials met the eligibility criteria (Fig. 1) Of those, 9,166 person-trials were excluded due to missing information on confounding factors. The final sample size was 1,379,708 nonunique individuals. There were 2,915 statin users. We then selected three nonunique individuals of the statin nonuser group (n = 8,525) per a use of statin group. Baseline characteristics of statin nonusers and users are shown in Table 1.

**Fig. 2:**
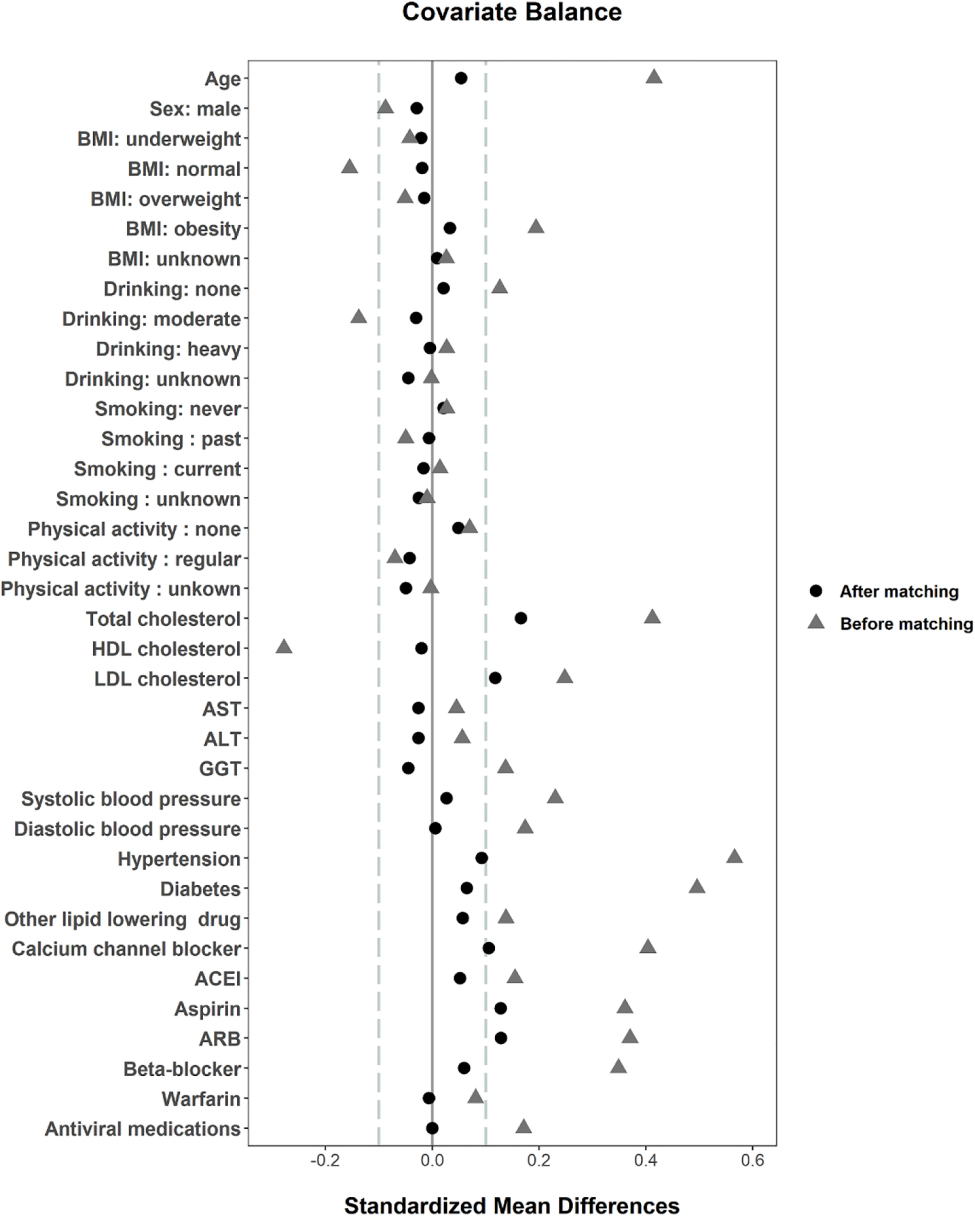
Covariate balance check

The mean (standard deviation) age of study participants was 55 years. Males accounted for 47% of participants (Table 1). All SMDs of differences between statin nonuser and statin user group were less than 0.1 except for total cholesterol, LDL-cholesterol, use of aspirin, angiotensin II receptor blockers, or calcium channel blockers (Fig. 2).

**Fig. 3:**
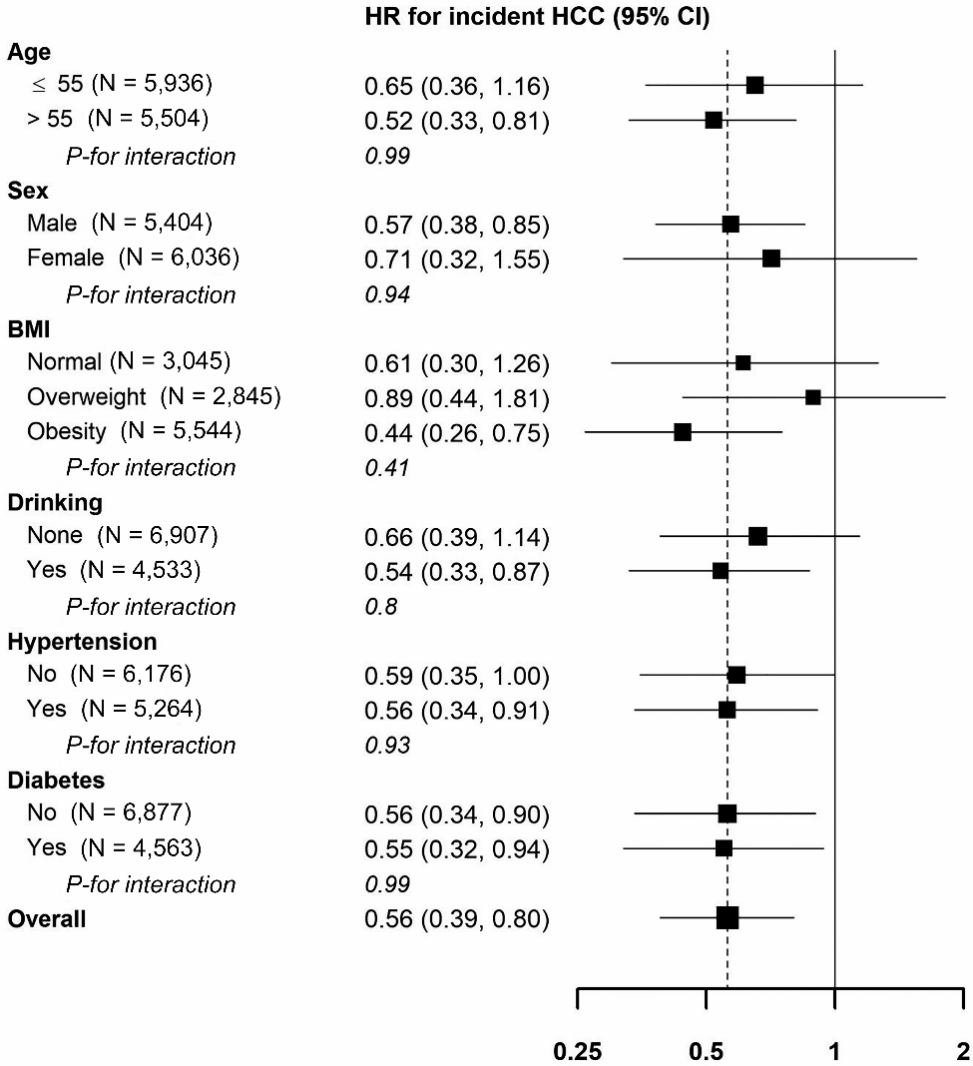
Subgroup analysis. Adjusted for total cholesterol, LDL cholesterol, use of calcium channel blocker, aspirin, and angiotensin II receptor blocker (covariates with SMD greater than 0.1)

During a median 5.8 years of follow-up, 207 participants developed HCC. Incidence rates of HCC in statin user group and statin nonuser group were 0.2 and 0.3 per 1,000 person-years, respectively (Table 2). The fully adjusted HR for incident HCC comparing statin user group to statin nonuser group was 0.56 (95% CI: 0.39 to 0.80). The association between use of statin and HCC was consistent in all subgroups analyzed (Fig. 3).

**Table 2:** Hazard ratios for incidence of hepatocellular carcinoma and mortality according to statin use

	No of case (100 persons year)	Crude HR (95% CI)	Adjusted HR^a^ (95% CI)
**HCC**			
Statin nonuser	170 (0.3)	Reference	Reference
Statin user	37 (0.2)	0.56 (0.39, 0.80)	0.56 (0.39, 0.80)
**Liver cancer or liver-related mortality**			
Statin nonuser	86 (0.2)	Reference	Reference
Statin user	17 (0.1)	0.57 (0.33, 0.94)	0.59 (0.35, 0.99)
**Extrahepatic cancer-related mortality**			
Statin nonuser	85 (0.2)	*Reference*	*Reference*
Statin user	34 (0.2)	1.13 (0.76, 1.68)	1.12 (0.75, 1.67)
**Cardiovascular disease-related mortality**			
Statin nonuser	42 (0.1)	*Reference*	*Reference*
Statin user	14 (0.1)	0.94 (0.51, 1.72)	0.96 (0.52, 1.79)
**All-cause mortality**			
Statin nonuser	302 (0.6)	Reference	Reference
Statin user	101 (0.6)	0.94 (0.75, 1.18)	0.93 (0.78, 1.11)

*HR* Hazard Ratio, *CI* Confidence Interval, *HCC* hepatocellular carcinoma

^a^Adjusted for total cholesterol, LDL cholesterol, use of calcium channel blocker, aspirin, and angiotensin II receptor blocker (covariates with SMD greater than 0.1)

There were 403 cases with mortality observed in this study. The most common cause of mortality was extrahepatic cancer (28%), followed by liver cancer or liver-related mortality (23%), cardiovascular diseases-related mortality (14%) and injury, poisoning and external causes related mortality (12%). Liver cancer or liver-related mortality rates in statin user and statin non-user groups were 0.1 and 0.2 per 100 person-years, respectively (Table 2). The fully adjusted HR comparing statin user group to statin nonuser group was 0.59 (95% CI: 0.35 to 0.99) for liver cancer or liver-related mortality, 1.12 (95% CI: 0.75 to 1.67) for extrahepatic cancer-related mortality, 0.96 (95% CI: 0.52 to 1.79) for cardiovascular disease-related mortality and 0.93 (95% CI: 0.78 to 1.11) for all-cause mortality (Table 2).

## Discussion

In this study, we found a lower risk of incident HCC in CHB patients who were statin users compared to statin nonusers. It provides strong support to the hypothesis that statins can be a chemo-preventive agent for the prevention of HCC development in patients with chronic HBV infection. Statins are mostly indicated for treatments of dyslipidemia. Treatments of dyslipidemia using statin do not exclusively rely on total cholesterol level. Multiple factors should be considered when starting statin therapy [24]. Awareness, treatment, and control rate of dyslipidemia are reported to be suboptimal in the general population, especially in patients with liver diseases [25, 26]. In previous observational studies that investigated the association between statin and HCC [8], CHB patients received statin therapy to treat dyslipidemia, not to prevent HCC development. Total cholesterol level is lower in patients with more severe liver diseases [27]. It is inversely associated with HCC risk [28]. Hence, statin use by indication (dyslipidemia) in previous observational studies is a great concern in that statin user group might have virtually different risks of HCC compared to statin nonuser group. To avoid this potential bias, we used total cholesterol level of 200 mg/dL or higher as an eligibility criterion. Also, by excluding participants with advanced liver disease and by performing propensity score matching of several important confounders for HCC including host factors (age and sex), social factors (drinking, smoking, and physical activity), severity of liver disease (AST, ALT, GGT levels, and antiviral therapy for HBV), and metabolic factors (BMI, total cholesterol, HDL cholesterol, LDL cholesterol, diastolic blood pressure, systolic blood pressure, hypertension, diabetes, other lipid lowering drug, antihypertensive medications, and aspirin), we could emulate CHB patients who have dyslipidemia and a similar risk of HCC, but differ only by statin therapy.

Another major strength of this study was the application of an advanced analytic method called trial emulation that accounted for measured differences between treatment arms, which precluded causal inference and reduced the issue of immortal-time bias. Previous observational studies used a time zero that violated condition. Some studies classified individuals as statin users based on statin use that took place before time zero (prevalent users), which might result in a selection bias [29]. However, the trial emulation method has been considered as one of the methods for making a balance of timing of eligibility between exposed and non-exposed groups and treatment assignment in observational analyses [23]. Thus, our analysis emulated a target trial of statin therapy initiation when participants without a history of hyperlipidemia were initially detected with greater than 200 of cholesterol at screening. To do so, we set time zero of our analysis to correspond with time zero in the target trial, that is, the time when both (1) eligibility criteria were met and (2) treatment strategies were assigned.

Several mechanisms could explain the chemo-preventive effect of statin. Statins can inhibit downstream products of the mevalonate pathway, mainly geranylgeranyl pyrophasosphate and farnesylpyrophosphate, which are crucial for malignant cell proliferation [30]. Reduced activation of proto-oncogenic transcription factor Myc with an involvement of cell mitochondrial membrane potential by statins has also been observed in HCC [31]. Statins might also exert anti-HBV activity by inhibiting cholesterol synthesis and HBV replication [32]. These pre-clinical evidences, together with findings from this study, strongly support anti-cancer effects of statin in CHB patients.

In terms of mortality, we have observed a decreased risk of liver cancer or liver-related mortality among individuals who use statins. However, no significant difference in the risk of all-cause mortality was identified between statin users and non-users. Upon analyzing cause-specific mortality, we found no significant risk reduction in extrahepatic cancer-related mortality or cardiovascular disease-related mortality among individuals who use statins, which are common causes of mortality in this population. This may explain why the benefit of statin in reducing liver cancer or liver-related mortality did not translate into a decreased risk of all-cause mortality. Furthermore, liver cancer-related death certificate codes (C22) do not differentiate between C22.0 (HCC) and C22.1 (intrahepatic cholangiocarcinoma) death. As a result, the observed benefit of statin therapy in reducing the incidence of HCC may have been attenuated in reducing liver cancer-related mortality.

This study has some limitations. The natural course of CHB is diverse and the risk of HCC differs significantly by different clinical phase (e.g., immune-active, immune-inactive, immune-tolerant) [33, 34]. The degree of liver fibrosis is another strong risk factor for HCC [35]. In this study, we excluded cirrhosis or severe liver diseases, and included participants with elevated serum cholesterol level. The characteristics of the studied cohort revealed that the proportion receiving antiviral therapy for HBV was very low (1%) with relatively low HCC incidence rates (0.2 ~ 0.3 per 1,000 person-years). Of note, serum cholesterol level is usually low for CHB patients with cirrhosis or advanced liver fibrosis [36]. Thus, low proportion of antiviral therapy, low HCC incidence rates, and elevated serum cholesterol level suggest that the studied cohort might be comprised of people in their immune-tolerant or immune-inactive phase without advanced liver fibrosis. However, as we lacked variables to assess the degree of liver fibrosis, this is only an assumption, and need further evaluation and validation using a cohort with information on clinical phase of HBV infection and degree of liver fibrosis. In this study, we used serum cholesterol level to identify patients who are potentially indicated for statin therapy. However, the indication or criteria to use statin treatment for dyslipidemia is not based on serum cholesterol level alone [37]. In brief, risk factors such as smoking, hypertension, age, family history of atherosclerosis and serum HDL-cholesterol level should be considered in addition to serum total cholesterol level or LDL-cholesterol level. We included those with elevated serum total cholesterol levels but may have included those who are outside of statin treatment indication. We also had to presume that all prescribed medications were taken by participants as prescribed, which might have overestimated the actual ingested dosage because some degrees of noncompliance are always expected. In addition, further investigations are needed to clarify whether intensity or type of statin (lipophilic or hydrophilic) is associated with chemo-preventive effect of statin, and what cut-off value of cholesterol level should be recommended to consider statin therapy in this population. Furthermore, information regarding side effects of statins had not been systematically collected. Lastly, the cohort was comprised of Koreans. Almost all Korean patients were infected with genotype C HBV [38]. Hence, our findings might not be generalized to different ethnicities or those infected with other HBV genotypes. Nevertheless, the large sample size, long follow-up duration, using cholesterol level as an eligibility criterion, and emulation target trial design are advantages of this study.

In conclusion, the present study showed that statin might have a benefit in preventing HCC in CHB patients with elevated cholesterol levels. Statin therapy may decrease the risk of HCC and liver cancer or liver-related mortality in this population. Our findings call for RCTs to assess the risk-benefit ratio of statins for chemoprevention of HCC in CHB patients. Until such data are available, active consideration of statin for CHB patients with elevated total cholesterol levels is needed.

## Data Availability

The datasets used and/or analysed during the current study are available from the corresponding author on reasonable request.

## Abbreviations

ALT: alanine aminotransferase
AST: cholesterol, aspartate aminotransferase
BMI: body mass index
CHB: Chronic hepatitis B virus
CI: confidence intervals
GGT: gamma-glutamyl transferase
HBV: hepatitis B virus
HCC: hepatocellular carcinoma
HDL: high-density lipoprotein
HMG-CoA: 3-hydroxy-3-methylglutaryl CoA
HR: hazard ratios
ICD-10: International Classification of Diseases, 10th Revision
K-NHIS: Korean National Health Insurance Service
LDL: low-density lipoprotein
PS: Propensity score
RCTs: randomized controlled trials
SMD: standardized mean difference
